# Retraction Notice to: Bone Marrow Mesenchymal Stem Cell-Derived Exosomal MicroRNA-126-3p Inhibits Pancreatic Cancer Development by Targeting ADAM9

**DOI:** 10.1016/j.omtn.2022.08.024

**Published:** 2022-08-22

**Authors:** Dong-Mei Wu, Xin Wen, Xin-Rui Han, Shan Wang, Yong-Jian Wang, Min Shen, Shao-Hua Fan, Zi-Feng Zhang, Qun Shan, Meng-Qiu Li, Bin Hu, Jun Lu, Gui-Quan Chen, Yuan-Lin Zheng

## Main text

(Molecular Therapy: Nucleic Acids *16*, 229–245; June 2019)

This article has been retracted at the request of the Editor-in-Chief and Academic Committee of Jiangsu Normal University (ACJSNU). ACJSNU informed the journal that in July 2020, ACJSNU became aware of PubPeer comments questioning Figures 3B, 3C, and 6C (https://pubpeer.com/publications/00DC0E3EE81793F83AC0EC80ACD256?utm_source=Chrome&utm_medium=BrowserExtension&utm_campaign=Chrome). As a result, ACJSNU launched an investigation and invited two independent referees to review the issues raised by PubPeer. Both referees agree that the paper displays signs of scientific fraud. In the meantime, ACJSNU requested that the corresponding authors of the paper provide the original experimental records and data for verification. However, the authors have failed to provide the requested documents and data to ACJSNU for examination. ACJSNU also noted that no surgeries are performed at the School of Life Science, College of Health Sciences, Jiangsu Normal University; it is unclear where the surgical specimens were obtained. Based on the investigation results, ACJSNU and the Editor-in-Chief believe that the paper has violated academic integrity and thus requested to retract this paper. Corresponding author Jun Lu disagrees with the retraction and stated that the data in question were obtained from a third party, which was not disclosed in the article.

